# Logic model for opioid safety in chronic non-malignant pain management, an in-depth qualitative study

**DOI:** 10.1007/s11096-022-01493-6

**Published:** 2022-11-25

**Authors:** Ayesha Iqbal, Roger Knaggs, Claire Anderson, Li Shean Toh

**Affiliations:** 1 Division of Pharmacy Practice and Policy, School of Pharmacy, University of Nottingham, Nottingham, NG7 2RD UK; 2 Primary Integrated Community Services, Unit H4 Ash Tree Court, Nottingham Business Park, Nottingham, NG8 6PY UK

**Keywords:** Chronic non-malignant pain, Community pharmacy opioid service, Logic model, Opioid optimisation, Pakistan

## Abstract

**Background:**

Opioids are commonly used for the management of chronic non-malignant pain in Pakistan; but there is a lack of literature around precursors or motivators in the use of opioids.

**Aim:**

The study holistically explored factors contributing towards the unsafe use of opioids and identifies strategies to overcome them.

**Method:**

Exploratory qualitative methods using interviews, focus groups and non-participant observational case studies were used. Interviews and focus groups were carried out face-to-face as well as virtually and observations were conducted in community pharmacies in Islamabad and Khyber Pukhtoon Khuwa province, Pakistan. Data were collected from 4 stakeholder groups; pharmacy policy makers (n = 11), people with chronic non-malignant pain (n = 14), doctors (n = 31) and community pharmacists (n = 36) by purposive critical case sampling method. Data were analysed inductively using reflexive thematic analysis and then deductively mapped to a social ecological framework. Non-participant observations were analysed using a cross case synthesis using explanation building technique. Data from all three methods were triangulated to develop a logic model.

**Results:**

Identified factors at macro (regulation), meso (social perceptions of pain and opioids) and micro levels (uncontrolled pain, self-medication, health literacy) and strategies are presented holistically and were used to develop a logic model for the prevention and mitigation of factors currently causing unsafe use of opioids.

**Conclusion:**

The study provides an in-depth view of factors contributing towards diversion of pharmaceutical opioids and can help guide national and international policy makers in their future initiatives to promote safe use of opioids in the management of chronic non-malignant pain in Pakistan.

**Supplementary Information:**

The online version contains supplementary material available at 10.1007/s11096-022-01493-6.

## Impact statements

- Identifies various strategies on various social ecological level leading or enabling safe use of opioids in Pakistan.
- Provides an overview of current factors contributing towards unsafe use of opioids and need-based evidence for development of novel community pharmacy services for the optimisation of opioids in chronic non-malignant pain.
- Provides a pathway/roadmap for safe use of opioids which might improve access to essential medicines like morphine for pain management under the Universal Health Coverage goals in Pakistan.

## Introduction

Chronic non-malignant pain (CNMP) is a big challenge in healthcare [1]. A systematic review of 28 low- and middle-income countries (LMICs) highlighted that 34% people suffer from chronic pain [2]. Pain relief is a basic human right [3] but most LMICs lack access to potent opioids due to poor regulation and unavailability of medicines [4]. Following the objectives of Universal Health Coverage which aims for “access to quality essential medicines and medicine services”, many LMICs are looking to increase access to opioids and provide better pain management options. Pakistan, a LMIC, is seeking to improve the safety of available medicines as well as improve overall access to medicines by introducing new legislative measures and regulating the dispensing of narcotics (opioids such as morphine, nalbuphine, fentanyl, tramadol) and other prescription medicines [5].

Opioids are commonly used to manage CNMP [6] but some people may develop problematic opioid use, which may increase the risk of opioid related morbidity and mortality. A recent systematic review (2021) reported that 36% (n = 55,647 patients) [7] people with CNMP used opioids unsafely, whereas a previous review (2015) highlighted 76% misuse of opioids [8]. Numerous risk factors can contribute to vulnerability to problematic opioid use related to prescription opioids. These may include individual, family, peer, social, and environmental factors, with genetically driven characteristics playing an important direct or indirect role within these domains. Individual risk factors may also include a prior history of substance use disorder, certain demographic features (such as younger age), more severe pain, and co-occurring emotional disorders [9]. Other personal and interpersonal risk factors such as divorce, unemployment, or irregular employment have also been reportedly associated with increased risk of opioid prescription misuse [10].

A survey conducted by the United Nations (UN) Office on Drug and Crime in 2013 showed an estimated 6.7 million adults in Pakistan (total population was 181.7 million in 2013) used opioids. Of these, 4.25 million used opioids unsafely and 1.6 million people reportedly misused prescription opioids [11]. Although the updated statistics in Pakistan about the usage of prescription opioids is lacking however, a 2018 study reported that from 2004 to 2015 a drastic increase in the trend of prescribing tramadol in developing countries including Pakistan. The same study also highlights Pakistani physicians’ high preference to prescribe tramadol to treat chronic pain in Pakistan [12]. Increased preference for tramadol prescribing is also reported in another study by Iqbal et al. [13] from Pakistan which reports (n = 740) that amongst the consumption of opioids in different pain types, tramadol was the most used opioid among all other opioids for CNMP condition like skeletal disorders [13]. In addition, a 2021 study from Pakistan shows that opioids such as tramadol, codeine and nalbuphine remain freely available over the counter in licenced pharmacies and medicine retail outlets without a prescription [14].

In this study, the term “unsafe use of opioids” encompasses irrational use of opioids as per World Health Organisation (WHO) standard definition, “irrational use of medicines implies that patients get medications inappropriate to their clinical conditions, doses not that meet their requirements for the desired period”[15] as well as misuse, inappropriate use, non-adherence, and/or self-medication with opioids which may increase the potential of opioid related harm.

Numerous studies have reported that social ecological factors might directly or indirectly influence the capability or trigger the motivation of individuals with CNMP to use opioids unsafely [16–18]. Thus, before improving access to potent opioids in Pakistan, it is imperative to understand factors affecting opioid medication use behaviour of people with CNMP.

### Aim

The study aimed to holistically explore factors promoting or contributing to the unsafe use of opioids in Pakistan and identify possible strategies to overcome them.

### Ethics approval

Ethics approval was obtained from Research and Ethics Committee School of Pharmacy University of Nottingham, UK no. 018–2019 on 16/12/2019 and Research Ethics Committee, Hamdard University Islamabad, Pakistan no. HU-ERC-19–400 on 05/12/2019.

## Method

### Study design

In-depth interviews, focus groups discussions and non-participant observation case studies were used in this exploratory qualitative study. The study follows the Consolidated Criteria for Reporting Qualitative Research (COREQ) checklist to provide transparency and rigour to this study.

### Participant recruitment and consent

**Fig. 1:**
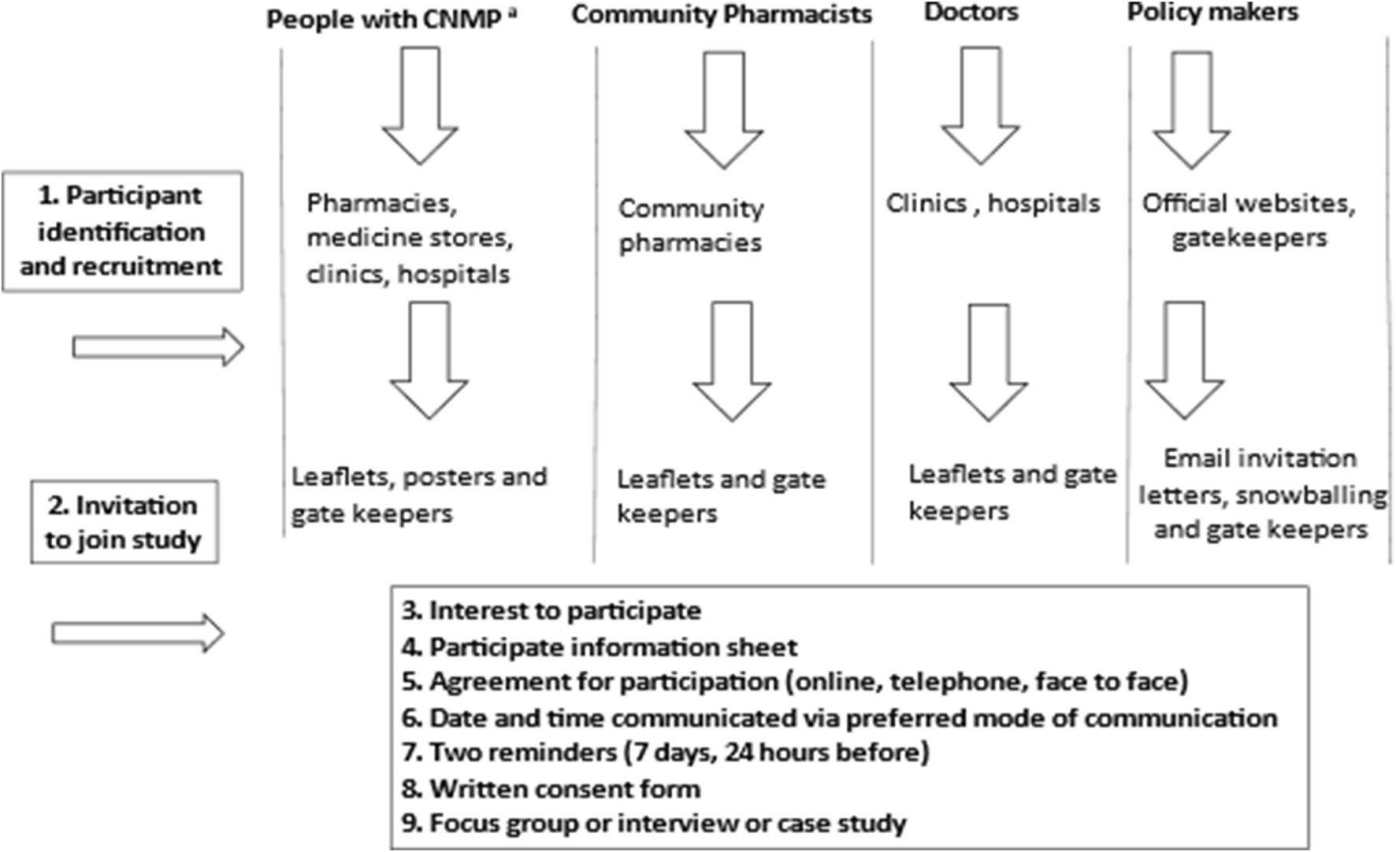
Participant identification, purposing critical case sampling and recruitment, **a** CNMP = chronic non-malignant pain

Participants were recruited using purposive sampling as shown in Fig. 1.

The inclusion criteria for people with CNMP, was continuous self-reported use of opioids for at least 1-year. Purposive critical case sampling was used to select pharmacies to conduct observations as well as to select participants for interviews and focus group discussions, to obtain relevant information. All participants were provided a participant information sheet. Written informed consent (or consent form via email for virtual methods) was obtained, before interviews, focus groups and case studies took place. Interviews were conducted virtually using telephones whereas Microsoft Teams® was used for focus groups. Data were anonymised by giving participants a study code.

### Data collection methods and duration

#### Interview guide piloting and pilot observations

A semi-structured interview guide was drafted in English after an extensive literature review and was checked for content validity and face validity by 2 experts (pain and qualitative researcher). The translated version in Urdu (native language) was validated by1 native speaker (face validity). The interview guide was revised for language simplicity and clarity, and the piloted, validated version was used for data collection (Supplementary electronic material-Appendix 1).

#### Interviews and focus groups

The interviews and focus group discussions were conducted in the native language (Urdu), therefore back translation was applied to assure accuracy in translation. The transcriptions were conducted by AI (Master’s in Pharmacy Practice) and were validated by a private translation company in Pakistan to ensure accuracy. The transcripts were not sent back to participants. Interviews were conducted with pharmacy policymakers and people with CNMP whereas 5 focus group discussions each were conducted with doctors and community pharmacists. Interviews and focus group discussions were conducted between December 2019-December 2020, face-to-face, at a mutually convenient location usually universities, offices and cafes. No other person was within the hearing range of interview/focus group conversations, except the researcher and participant(s), to ensure confidentiality of data sharing, not even (any) accompanying family members. After March 2020 (due to the COVID-19 pandemic), 5 interviews with people and 4 with policy makers were conducted virtually using mobile phone, whereas 2 focus group discussions with doctors and 1 focus group with pharmacists was conducted via Microsoft Teams® (USA). All interviews/ focus group discussions were audio recorded and transcribed in the same manner regardless of whether they were collected virtually or face-to-face.

#### Case studies

Case study observations were carried out in 6 community pharmacies between September–November 2020. All activities involving opioid medications were recorded by continuous note taking in shorthand format using a checklist (Supplementary electronic material -Appendix 2) designed for the study.

The researcher’s perceptions of ongoing opioid medicine related events were recorded as “impressions” so they could be differentiated from the rest of the empirically observed notes. All non-formal conversations were drafted as communications. Thus, direct observations field notes, informal conversations and researcher impressions were drafted as case reports and have been presented as vignettes in this study. The vignettes provide an overview of a theoretically identified phenomenon in real life context and helped in knowledge contextualisation during data triangulation.

All interviews, focus group discussions and observational case studies were conducted by AI as part of her PhD work.

### Data analysis

**Fig. 2:**
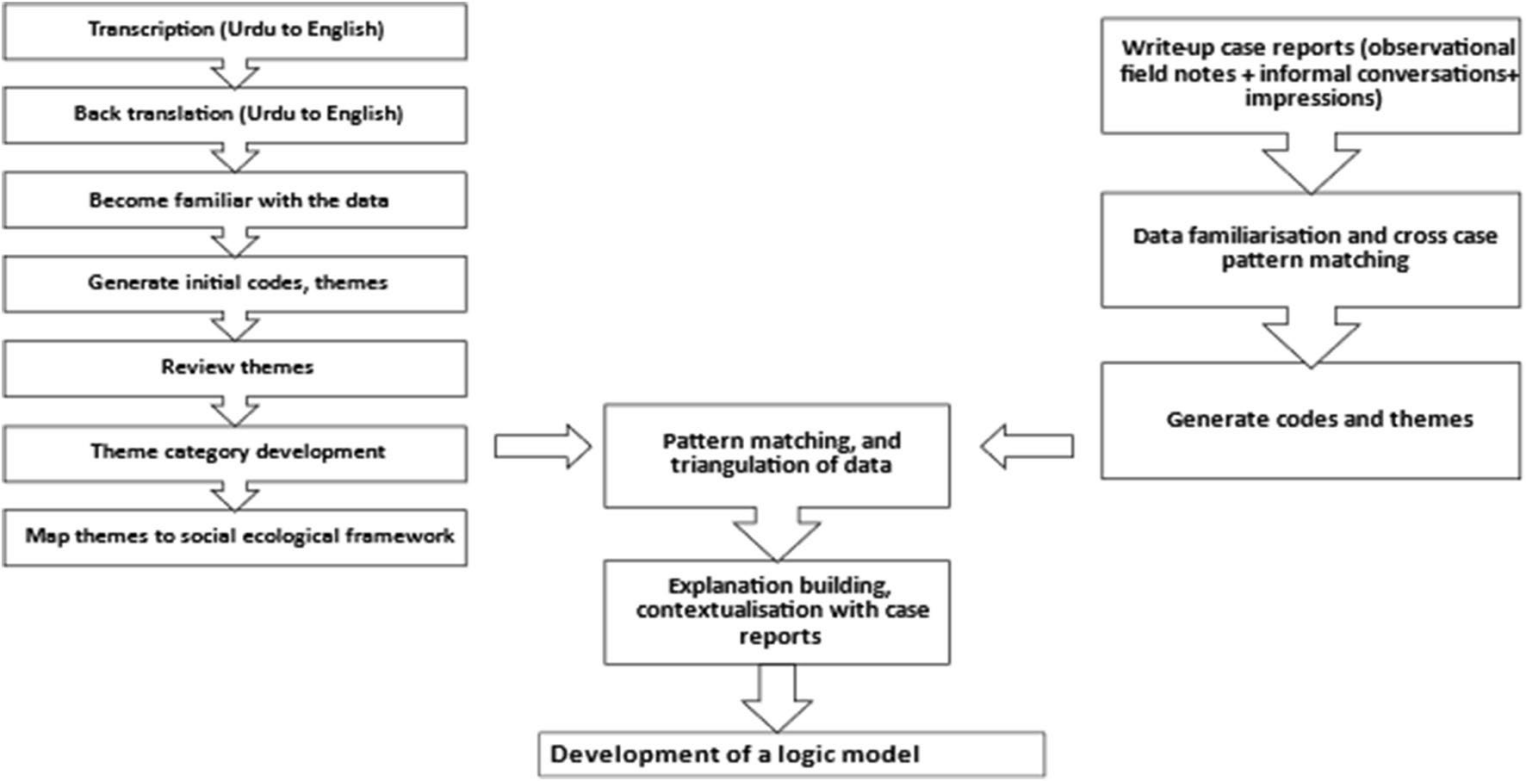
Inductive reflexive thematic analysis (RTA) followed by deductive mapping to social ecological framework

Analysis of case reports and interview/focus group discussions transcripts was undertaken using the reflexive thematic analysis [19, 20] electronically, utilising NVivo 12® (QSR International, Melbourne, Australia) as shown in Fig. 2. Themes and subthemes were then deductively mapped to the Social Ecological Model [21]. No analytical differences were observed between face-to-face and virtually collected data. The analysis was conducted by AI (reflexive thematic analysis) and thus other authors CA, RDK and LST sense-checked data interpretation, rather than introducing or developing a consensus on coding reliability [22].

The case studies were analysed using a cross case synthesis (Fig. 2) using explanation building technique [23], which supported in providing triangulation and contextual explanations for different observed phenomenon(s) and helped in identifying corresponding strategies. The Social Ecological Model is particularly well suited for complex multi-faceted factors and can help present a holistic view of the individual relationships, community, system and societal factors. Using process mapping and data visualisation techniques, the factors that may impact the use of opioids by a person with CNMP in Pakistan and may contribute towards unsafe use of opioids and identified strategies are presented in the logic model. It must be highlighted that these pathways do not show causality, but merely present the identified results in a logical way for ease of understanding. This model logically collates all evidence identified using the guiding concepts of theory of change [24] and realism [25] and shows that certain strategies might be able to overcome current deficiencies contributing towards unsafe use of opioids and might achieve desired change of achieving outcomes over time [26].

### Data trustworthiness and reflexivity

AI received training in conducting qualitative research and data analysis prior to data collection. The analytical review of codes and corresponding themes by other authors helped provide trustworthiness and minimised self-bias. Data collection continued even after data saturation had been achieved in interviews and focus group discussions, to develop a robust and valid understanding of the study phenomenon. In an attempt to reduce the Hawthorne effect [27], the staff within pharmacies were not told how many days the researcher AI would be observing. In addition the observations continued after data saturation was reached in case studies to reduce the Hawthorn effect as well as to capture any new data. Data saturation was supposedly achieved in the study when no new information was being obtained in subsequent interviews, focus groups and case studies. The data for each stakeholder as well as case studies have been provided in supplementary electronic material- Appendix 3.

## Results

A total of 98 stakeholders participated in the study out of which, 38 were female. The total number and overall demographics of participants are presented in Table 1 below, whereas the detailed characteristics of people with CNMP are in the Supplementary electronic material- Appendix 4.

**Table 1:** Stakeholder demographics

Stakeholder group	Policy makers	People with CNMP	Doctors	Community pharmacists
Gender (female %)	0% n = 11 (male) n = 0 (female)	50% n = 7 (male) n = 7 (female)	48% n = 16 (male) n = 15 (female)	33% n = 24 (male) n = 12 (female) Case studies 66% n = 2 (male) n = 4 (female)
Age range in years (mean) Standard deviation (SD)	31–58 (47.90) SD = 8.65	31–75 (58.92) SD = 11.55	24–74 (31.12) SD = 11.21	24- 38 (28.47) SD = 3.23
Years of experience* (mean) standard deviation SD	20–35 (14.90) SD = 8.04	1.5–10 (6.78) SD = 5.29	6–45 (5.87) SD = 10.20	1–16 (2.87) SD = 2.71
Response rate % (approached /declined)**	73.34% 15/4 (Agreed to participate but refused due to time commitment)	77.78% 18/4 (Agreed to participate but did not respond after initial agreement, or failed to show-up for interview)	78.95% 38/8 (Agreed to participate but did not respond after initial agreement, or failed to show-up for FGs discussion)	85% 40/6 (Agreed to participate but did not respond after initial agreement, or failed to show-up for FGs discussion)

*= For people years of continuous opioid use

**= People who had agreed to participate but due to other time commitments could not make the interview or focus group discussion

Interviews lasted from 50 min to 1 hour and 30 min whereas focus group discussions took 1.15  to 2 hours on average.

**Fig. 3:**
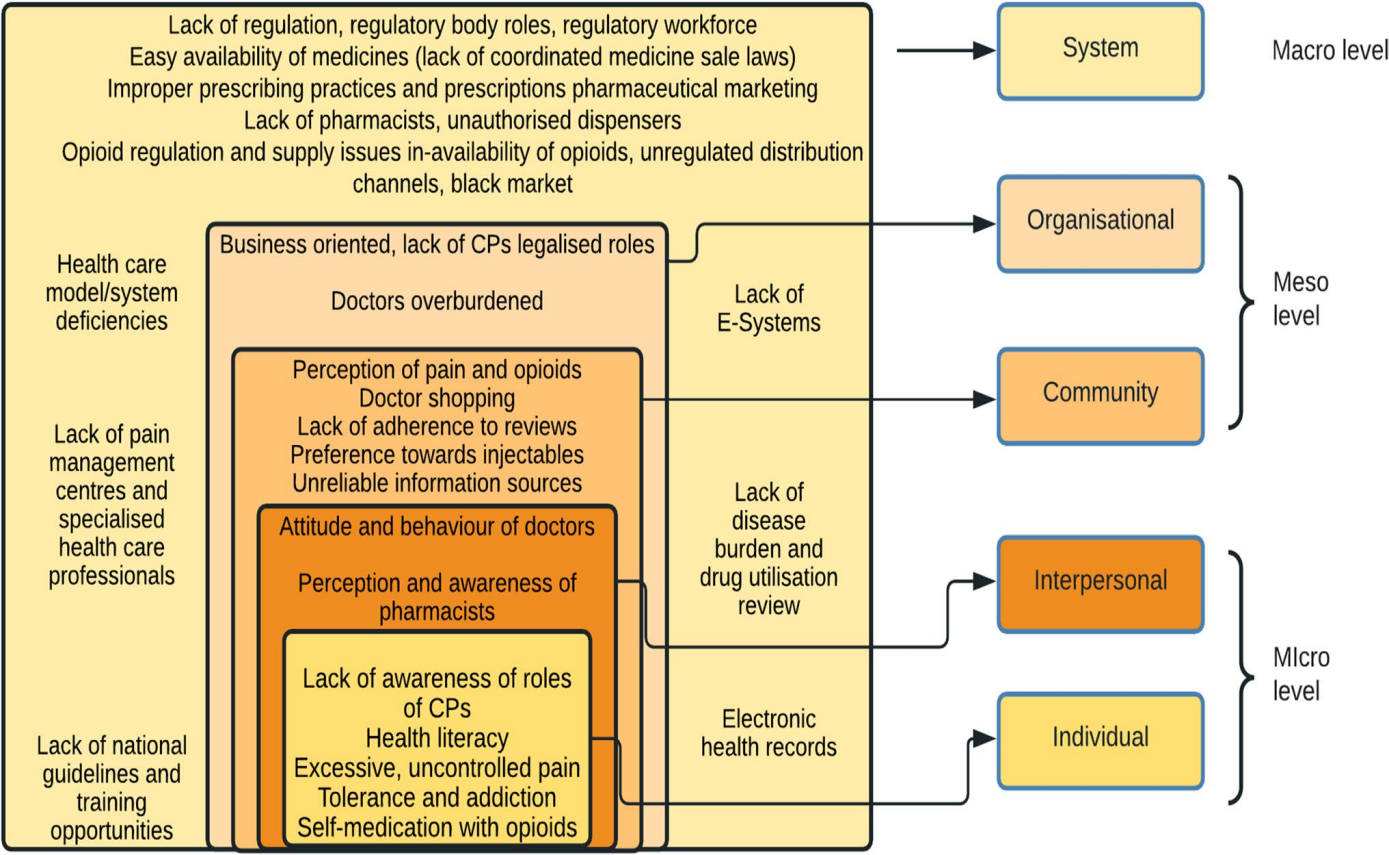
Identified factors contributing towards unsafe use of opioids mapped to the Social ecological model

A total of 240 hours (40 hours/case) were observed during a 6-week period of non-participant observational case studies in 6 community pharmacies. Factors identified across the 5 levels of the social ecological model as shown in Fig. 3 are now considered below.

### System

#### Regulation

Pakistan has an inadequate regulatory workforce to control the unsafe use of opioids. Despite being under regulation opioids remain freely available without prescription.

"Currently the regulators (drug inspectors) are so scarce that there are maybe 1 or 2 people in the whole district... How can they enforce the proper rules in all shops?" Policy Maker 8 (also see Vignette 1, in Table 2)

**Table 2:** Vignettes observed in case studies providing contextual explanations or actions of phenomenon of interest, theoretically perceived in real life settings under different themes and sub-themes

Themes	Subthemes	Vignette
System	Regulation	Community pharmacies were more strictly following the law, where there was a high chance of inspection via drug inspectors and vice versa. The inspection by regulators was found to be location dependent, and inspectors were observed to visit community pharmacies in easy, prominent and high traffic locations, However, remote areas or quiet areas within a city were easily bypassed. Vignette 1 In all cases, all prescriptions had brand names of the opioid medications, and many of the prescriptions were found to have therapeutic duplication of opioids as well as other analgesics. People arriving at pharmacies, sometimes filled more than two prescriptions, from different doctors, having similar opioid medications (doctor shopping). Polypharmacy with analgesics was common. Vignette 2
Health care system deficiencies	E-Systems	Instances were recorded where prescriptions arriving at pharmacy shop had medicines added in a different ink, potentially hinting “illegal prescription adulteration/addition” , which highlights the disadvantage and limitations of manual prescribing systems and prescriptions. Vignette 3
Organisational	Business oriented medicine outlets	The pharmacies were found to be dispensing as per prescription, changing medicines according to a person’s preference, dispensing when patient asked a particular medicine (without a prescription), advised/prescribed medicines (where pain was discussed without taking any particular name) and referred to doctors. All activities remained in line as “not to compromise” the potential sell as well as improving patient/customer experience, to ensure their return. Vignette 4
Community, social and cultural	Awareness of medicines	Pharmacists stamping prescriptions to infer opioid dispensing, was negatively perceived by people, who were observed to complain outright that other “neighbouring pharmacies were not making their life more difficult” and complained "they would have to again go back to doctors and pay them fee, to get this prescription" because they (pharmacists) *“*ruined it*”* and "they could have used it many times". Vignette 5
Interpersonal level	Perception of pharmacists	Most people proceeded towards the salesperson in pharmacies, ask medicine related queries (mostly dose, frequency and food) and made the purchase and went away, without enquiring or engaging with pharmacist for medicine information. However, a few people who (possibly) knew or had a past conversation with the pharmacists specifically asked the salespersons to speak to pharmacists and discussed their opioid medicines. Vignette 6
Individual people with CNMP	Health literacy	Instances where pharmacists tried to engage people themselves and tried to discuss their medications were met with mixed reactions where some would agree to talk and were interested in listening, however, some were reluctant and refused. People without a prescription (self-medicating, self-managing) were more difficult to convince to go back to doctors for review. However, they were receptive to pharmacist suggestions and advice about signs and symptoms of opioid related harm. Vignette 7

Medicine distribution channels inside Pakistan are poorly regulated.

"Even if an item is banned, that can also be found there... imported black market items... everything is available... These markets supply these medicines all over the country... They are found in every city... No check is in place..." Pharmacists Focus Group 3 P8

It was highlighted that under the new drug rules opioid dispensing can only be done by qualified pharmacists. However, as there is no pharmacist in most retail outlets, medicines are dispensed by pharmacy assistants and salespeople.

"The major problem is availability of pharmacist... when the pharmacist is not present, how can they control..." Policy Maker 4

Policy makers emphasized the regulatory workforce should be sufficient to ensure that opioids are distributed and dispensed according to regulations for prescription only medicines, in the presence of qualified pharmacists.

It was highlighted that pharmaceutical marketing to prescribe opioids might be playing a significant role in contributing towards unsafe prescribing practices.

"In this whole scenario... we are missing a very vital point... the pharmaceutical marketing... the market influences a lot of medicine prescribing...they control in a way with their incentives which drug should be more prescribed, this is unethical but unfortunately happening…." Doctors Focus Group 1 P1

Doctors, pharmacists and policy makers highlighted that most prescriptions do not conform to legal requirements due to lack of regulatory checks on physician prescribing practices, which may promote unsafe prescribing of opioids. (Vignette 2, in Table 2).

### Health care system deficiencies

#### Access to pain management

Pakistan lacks access to adequate pain management facilities, specialists and opioid medicines.

"From last 10 years or so, I think there are 2 centres which I know, which are specialised for pain management... there are no CNMP centers…" Doctors Focus Group 5 P6

"Currently… for long term pain management... there are only 2 products left... Tramadol… or nalbuphine… we have to rely only on these 2 medicines…" Doctors Focus Group 4 P1

The combination of these factors was perceived to lead people to visit non-specialist physicians or overuse available opioids, which could substantially increase the risk of opioid related side effects in the long-term management of pain as well as affect the quality of care.

#### E-Systems

Pakistan also lacks an integrated electronic health management system (E-Systems) and a pharmacist shared:

"We do not even know how many people experience these undue effects and ADRs... the reporting systems are missing... the situation in patient safety is terrible right now..." Pharmacists Focus Group 2 P5 (also see Vignette 3, in Table 2)

Most stakeholders emphasised that the government needs to invest in developing E-Systems and supportive infrastructure to promote the safe use of medicines, which might  facilitate patient data sharing across health care professionals and improve patient safety.

### Organisational

#### Business oriented medicine outlets

Pharmacists, policy makers and doctors highlighted the profit-making intentions of medicine outlet proprietors, and shared:

"Even if the doctor writes the medicine for 1 month e.g., (but) if the patient asks for 3 months of dose, they will gladly give, because, if they made profit of Rs 100 from 1 box, now the profit is Rs 300… there are no consequences of dispensing it more, who will ask them?... See there is a huge flaw... in the system that is causing medicine misuse..." Doctors Focus Group 2 P6 (also see Vignette 4, in Table 2)

#### Doctors overburdened

Pharmacists and doctors also shared that prescribers’ consult large number of patients in clinics and hospitals, which affects their ability to; engage in safe practices as well as to counsel patients for subsequent medications reviews, discouraging self-medication and perform risk assessment for opioids.

"There will be 4 doctors sitting in 1 room... they will be seeing their patients, there is so much (incoming) information.... I am speaking about the OPD (outpatient departments) clinics in the government hospitals... where majority of the people in this nation are going to seek health care..." Doctors Focus Group 1 P5

All stakeholders perceived that providing opioid medicine information is an essential parameter to ensure opioid medicine adherence in people.

"We need pharmacist in medicine stores to educate people for rational use and make sure they use it as intended, before giving them more (new, potent) medicines…" Doctors Focus Group 5 P6

### Community, social and cultural

#### Awareness of medicines

Poverty, lack of proper health facilities in rural areas and lack of health literacy might be contributing towards unsafe opioid use. People also engage in self-medication or demonstrate non-adherence to prescribed opioid regimens as well as avoid subsequent medical reviews due to lack of awareness.

"So, the general concept of the patients, is that they consider this (opioid) medicine like paracetamol that this is a normal pain medicine... Tramadol is still treated the same... just like an over the counter analgesic..." Pharmacists Focus Group 5 P1 (also see Vignette 5, in Table 2)

#### Doctor shopping

All stakeholders shared that “doctor shopping” is common in people with CNMP due to uncontrolled pain or after they develop tolerance.

"I am taking these medicines for years now, medicines are not effective anymore… that is why I visit different doctors, maybe they can help" Patient 6

Doctors and pharmacists shared that most people do not understand the risks associated with taking more opioids (increased dose or frequency) or duplicating them (polypharmacy), which increases the risk of adverse events (Vignette 2)  

#### Opioid injections

Pharmacists and doctors shared that a common social perception in Pakistan is, that people prefer obtaining injections from unauthorised personnels.

"I am talking about villages over here... the people leave their houses... with a set mind, that we have to go to that “doctor” (quack, dispenser)... and they will administer an injection..." Doctors Focus Group 4 P1

This social perception translates into increased opioid injection usage by both those who demand an injection and those who unlawfully administer them and substantially increase the risk and harm associated with injectable opioids.

Policy makers suggested improving social awareness about diseases and medications might motivate people to use their medicines more cautiously and the government should target public awareness about opioids, regulate dispensing and strictly monitor the unsupervised and unlawful administration of opioids and promote safe use of medicines.

### Interpersonal level

#### Attitude and behaviour of doctors

People with CNMP contributed that doctors exhibit empathy based on social class of the people. A person with CNMP shared:

"Nobody tells us the benefit or the side effects of the medicine.... not doctor even... if I ask, they will get angry... so I do not ask... when they look at me, they think I am illiterate... so they do not think me as worthy to answer..." Patient 1

However, doctors shared a different view and a doctor highlighted:

"In this country, people believe medicines (opioids) are needed… if we refer them for physiotherapy... and tell to use paracetamol... they will not even leave our office... they actually demand… medicines (opioids)..." Doctors Focus Group 3 P4

Doctors emphasized, lack of transparency in communications with patients, and the refusal to comply to unnecessary and unjustified opioid demands, usually impacts the physician–patient relationships. This may precipitate uncertainty in physicians’ professional advice, which can potentiate non-adherence as well as self-medication.

#### Perception of pharmacists

People with CNMP and pharmacists shared that even in those locations where community pharmacists are present, most people while purchasing opioids do not ask for additional information because of lack of awareness about the pharmacist’s role in medicines information, counselling and review.

"Also, our people... they're not even aware to ask this medicine information before buying the medicine..." Patient 3 (also see Vignette 6, in Table 2)

Stakeholders collectively agreed that the role of pharmacists is integral to promote safe use of opioids and developing specialised roles in opioid safety and improving public awareness about such roles can be beneficial for people with CNMP using opioids. 

### Individual people with CNMP

#### Uncontrolled chronic pain

All stakeholders shared that people with CNMP tend to increase the dose or frequency of medicines by themselves according to pain intensity, which could eventually lead to unsupervised use of opioids and may lead to opioid use disorder. A person with CNMP shared they might intentionally overdose opioids because:

"I think pain is a thing which does not let you forget... I am tempted... to overdose... or sometime take the medicine earlier than the scheduled time... just to avoid the pain getting worse..." Patient 10

#### Health literacy

Doctors and pharmacists highlighted that people might be taking a cocktail of medicines including complementary medicines without understanding the potential of harm and may advise each other regarding medicines or share medicines without understanding any synergistic effects, which may result in drug interactions and remain at high risk of opioid related adverse effects due to health illiteracy.

"Mostly they are uneducated... they do not agree to us... so they constantly keep on using the medicines... specially these addictive medicines" Pharmacists Focus Group 1 P1 (also see Vignette 7, in Table 2)

#### Mental health and other conditions

Policy makers, and doctors emphasized that people with CNMP with sleep disorders or mental health issues might be at a higher risk of overusing opioids because of possibly developing addiction or longing for the euphoric or sedative effects of opioids.

"So, when she (aunt) takes the medicine, she is happy... she is good... but when she leaves it... she gets very moody and cannot sleep... and has too much pain..." Patient 2

Stakeholders shared contrasting views, where pharmacists and people with CNMP highlighted that intentional prescription opioid abuse is not common in Pakistan. However, doctors and policy makers contributed that addiction with prescription opioid medicines such as nalbuphine and tramadol, is common especially in upper educated elite class where they escape the stigma of using illegal substances of abuse (e.g., heroin) but continue to obtain euphoria from opioids.

"Opioid misuse is a problem in Pakistan definitely. I have seen young patient who are addicted to opioid derivatives such as tramadol, nalbuphine and demand injection from healthcare professional without any indication…" Doctors Focus Group 5 P5

"If it’s a medicine there is no social pressure, yet it can provide euphoria…" Policy Maker 4

### Logic model for safe use of opioids

**Fig. 4:**
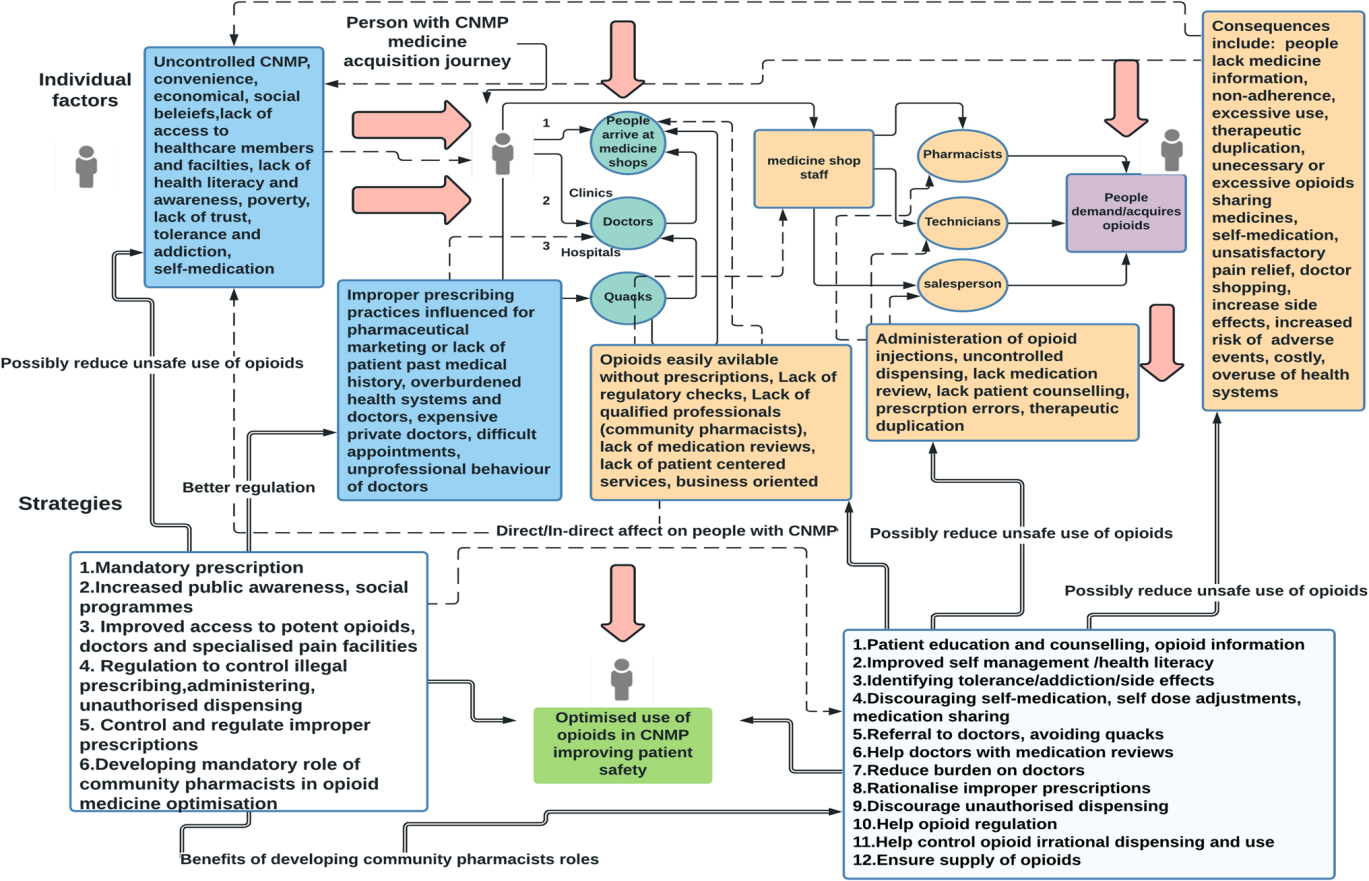
Logic model—factors and corresponding strategies to safely use opioid medications in people with CNMP

The identified factors were collated as a logic model (Fig. 4) that presents a diagrammatic representation of a sequence of actions and factors in the journey of a person with CNMP to acquire opioid medicines. The logic model also explains strategies identified by stakeholders that could counter the actions and factors contributing to opioid related harm.

Strategies at the system level include mandating and strictly enforcing supply of opioids only on prescription, increased public health awareness and literacy, improved access to potent opioids for adequate pain management, doctors and specialised pain facilities. Other system level strategies include control and regulation of, unlawful dispensing, prescribing and administration of opioids as well as ensuring conferring to standardised prescribing. Other strategies targeting multilevel factors include improving patient awareness, counselling and monitoring adherence with therapeutic regimes, medication reviews, discouraging self-medication and self-dose adjustments as shown in Fig 4.

## Discussion

### Statement of key findings

This is the first study to comprehensively explore and identify system, social, environmental, interpersonal and personal factors contributing/promoting the unsafe use of opioids in a LMIC, Pakistan and identifies strategies to overcome them as a logic model. The majority of the factors identified in the study such as uncontrolled pain, socio-cultural influences, sharing of opioids, over prescribing of opioids, influence of pharmaceutical advertising and inadequate regulation have been also highlighted in a US study using similar theoretical underpinning [28]. Although the factors identified in both studies are similar but they contextualise strategies to overcome these factors according to their respective socio-political environments. Other factors in the study such as, easy availability of medicines, preference for injectables, patient non-adherence to treatment regimens, lack of regulation, untrained or unlicensed staff administering and dispensing opioids in medicine outlets are similar to other studies reporting misuse of opioids or opioid related harm [29–31].

### Interpretation

The study highlights that fear of misuse and diversion of opioids by policy makers has led to lack of access to potent opioid analgesics leaving people in Pakistan suffering from chronic pain as well as facilitating, overdose or overuse of available opioids. Similar findings have been reported by studies in other LMICs [32] and lack of effective pain relief remains a major violation of UN sustainable development goal regarding good health and well-being.

US studies report that health policies aimed at controlling prescription opioid misuse, although demonstrating decreased misuse and diversion, have provided indirect motivation for people with opioid use disorder to shift to illegal opioids, thus failing overall to decrease opioid-related mortality [33]. Pakistan already has a very high number of heroin addicts amongst Southeast Asian countries [34], therefore it is imperative that policy makers be cautious and vigilant. Future studies should evaluate the possible impact and unintended consequences of enhanced opioid regulation for potent opioids. A possible meso level strategy, highlighted in this study was that developing the role of community pharmacists in opioid medicine optimisation could offer huge health system benefits in terms of controlling opioid misuse and diversion as well as promoting public safety. Community pharmacists may also be helpful in overcoming many factors at multiple levels (micro, meso and macro) identified within the logic model as well as facilitate the implementation of some of the proposed strategies and promote and enable safe use of opioids. Thus policy makers in Pakistan should investigate developing the extended role of community pharmacists in opioid optimisation.

The study findings highlighted that people in Pakistan can visit as many physicians as they want, which could result in polypharmacy, therapeutic duplications as well as significant interactions and may exacerbate unsafe use of opioids in people with CNMP. It is important to acknowledge that doctor shopping was one of the biggest contributors to the opioid crisis in US [35]. The precursors for doctor shopping in Pakistan are different from US because people doctor shop, because of unresolved CNMP, mistrust in prescribers or to get a confirmation of diagnosis and then use all the prescribed medications from all doctors, resulting in increased opioid usage whereas in US, people doctor shopped to obtain more opioids as they are likely to be unavailable without a prescription [36]. Thus, despite being different, contextually doctor shopping in both countries can lead to increased acquisition of opioids and may increase the utilisation of opioids by people with CNMP, and thus the chances and consequences of opioid related harm remain real. Additionally, this study reports that in Pakistan, people are not just doctor shopping [37, 38] but they also might engage in “pill shopping or pharmacy shopping” due to easy availability of medicines without prescriptions as well as lack of electronic prescription or dispensing records.

Additionally, opioid overprescribing by doctors, commonly known as “pill mills”, has contributed to opioid-related mortality and morbidity in the US [39] this was also identified as a factor in this study. Thus, a proposed macro level strategy would be to introduce generic prescribing, which might reduce the pharmaceutical promotional activities and incentivisation to prescribe their specific brand, along with Pakistan investing in E-Systems to facilitate collating opioid drug utilisation data, flag significant opioid related adverse drug events and mortality, as well as improve the accountability and transparency of prescribing and dispensing. Other studies also report the benefits of E-Systems in identifying opioid misuse and diversion and can be utilised for health surveillance and contribute to safe use of opioids [40].

It is important to acknowledge that it might be challenging to distinguish the pathway of different strategies proposed in this study at different social ecological levels of the health care system and evaluate their impact independently, as all strategies eventually target promoting public safety and optimising the use of opioids in people with CNMP.

### Further research

There appears to be a paucity of studies, both qualitative and quantitative and future studies in LMICs should target the use of opioids available for the management of CNMP, opioid drug utilisation data, opioid use disorder, satisfaction and adherence of patients with prescribed opioid medicines, as well as research the analgesic relief obtained with the use of current opioids in the management of CNMP. Future studies should also carefully evaluate the strategies identified in the study as well as the role of community pharmacists in optimising the safe use of opioids.

### Strengths and weaknesses

The study adopted a broad, holistic approach which helped identify possible underlying factors in social ecological levels and helped contextualise their corresponding strategies to improve opioid safety.

A methodological limitation was that case studies did not include medical stores, where pharmacists are not present, which could have overlooked some factors contributing to unsafe use of opioids. However, the pharmacies were purposefully chosen where pharmacy allied staff were involved in opioid dispensing without the involvement of pharmacists, thus the case studies might have managed to capture factors relating/contributing towards unsafe use of opioids due to non-pharmacist dispensing, therefore this limitation has been accounted for.

## Conclusion

Unsafe use of prescription opioids is influenced by a complex interplay of individual factors, interpersonal relationships, societal and system factors. This study presents a logic model, which proposes strategies that could help optimise the safe use of opioids as well as help improve access to opioids for the management of CNMP in Pakistan.

## Supplementary Information

Below is the link to the electronic supplementary material.

Supplementary file1 (DOCX 31 KB)
