# Supplementary material for: Logic model for opioid safety in chronic non-malignant pain management, an in-depth qualitative study
Source: Int J Clin Pharm. 2022 Nov 25;45(1):220–32. doi: 10.1007/s11096-022-01493-6 (PMC9702900; doi:10.1007/s11096-022-01493-6)
Supplement: Supplementary file 1 — Supplementary file1 (DOCX 31 KB) [file 11096_2022_1493_MOESM1_ESM.docx]

**Supplementary electronic material**

1. **Title:**

**Title: Logic model for opioid safety in chronic non-malignant pain management in Pakistan, an in-depth qualitative study**

**2. Full names of the authors;**

Ayesha Iqbal1*, Roger Knaggs1,2, Claire Anderson1, Li Shean Toh1

**4. Author's institutional affiliations:**

1 Division of Pharmacy Practice and Policy, School of Pharmacy, University of Nottingham, NG7 2RD, Nottingham, United Kingdom

2 Primary Integrated Community Solutions, Unit H4 Ash Tree Court, Nottingham Business Park, Nottingham NG8 6PY, United Kingdom.

**Corresponding author:**

Ayesha Iqbal

email: [ayesha.iqbal@nottingham.ac.uk](mailto:ayesha.iqbal@nottingham.ac.uk)

**Appendix 1**

# 1. Interview guide for people having CNMP

The semi-structured approach shall be used and emphasis will be given to the below subtopics in the context of understanding the norms and expectations of people with CNMP when they visit and interact with prescribers and community pharmacists, buy opioid medicines, and use opioid medicines.

**Pain management and health care related:**

- Can you share your experience/ How do you feel about the current pain management options available to people in Pakistan?
- Can you share your pain management experience and journey?

**Opioid medicine related:**

- Can you share in your words experience of using opioids in the management of CNMP?
- What do you think about the attitudes of the community pharmacist and prescribers towards your pain management?
- Can you please share, your current journey of using opioids, and any factors that helps you, use your medications more effectively or feel as barriers towards using medicines safely?
- Where do you normally get your medicine information/medication review, and would you like to share your experience receiving opioid medication information?
- What do you think is needed in the future so that people can get the best benefits of opioid medicines, without experiencing any, avoidable side effects and harms, while using opioids (optimization)?

**Interdisciplinary health teams:**

- How do you feel prescribers and community pharmacists can work together (as a team) to help manage pain by opioids? Any thoughts?
- How do you feel if a pharmacist has a concern/suggestion about your opioid therapy and usage and engages you in counselling session or if communicates the new plan with your prescriber?

**“If they highlight positive views about community pharmacy based pain management and opioid optimisation services, otherwise ask, opinions, views, suggestions or strategies to optimise the use of prescriptions opioids (general)”**

**Community pharmacist related (major priority)**

- What in your perception is the role of community pharmacists in helping you manage opioid medications for chronic pain?
- What do you think about the current community pharmacist services for pain management?
- Where do you see the community pharmacist’s future role to help people using prescription opioids in managing chronic pain to maximise the benefits and reduce harm?
- What should be the key attributes of a satisfactory review service in community pharmacy to avoid prescription misuse and optimise opioid therapy in CNMP?

“**Ask if there** a**nything they would like to add and highlight which wasn’t addressed during interview”**

2. Interview guide for community pharmacists

The data will focus on obtaining community pharmacist perception on current use of opioids in the management of CNMP and possible services and strategies for optimization of opioid therapy.

**Health system and pain management:**

- Can you please share your views on current use of opioids in the management of chronic non-malignant pain?
- Can you share your views on chronic pain management in Pakistan?

**Policies and legislation:**

- What are your views regarding current opioid regulation (schedule-G) and access to opioids for the management of CNMP?
- What guidelines and information do you follow while reviewing patient opioid medicine regimens?
- What do you think is needed in the future in the optimization of prescription opioids?

**“If they highlight positive views about community pharmacy-based pain management and opioid optimisation services), otherwise ask, opinions, views, suggestions or strategies to optimise the use of prescriptions opioids (general)”**

**Perception of the role of community pharmacist in optimization of opioid therapy:**

- Do you think of any services pharmacist could provide for opioid therapy optimization?
- Are community pharmacists playing any role in patient education, counselling, review and monitoring (for misuse and addiction) in prescription opioids in CNMP?
- What do you think the key attributes of care in a service provided by community pharmacist to optimise opioid therapy should be? Any barriers?

**Any barriers?**

- What is your experience when you review patient’s opioid medicine in CNMP? (prescription related)
- What is your experience when you counsel, educate, and provide medicine information to people suffering from CNMP to optimize their opioid use? (patient oriented)

“**Ask if there** a**nything they would like to add and highlight which wasn’t addressed during focus group discussions”**

# 3. Interview guide for doctors

The data will focus on obtaining doctors perception on current use of opioids and possible services for optimization of opioid therapy.

**Health system and pain management:**

- Can you please share your views on current use of opioids in the management of chronic non-malignant pain?
- Can you share your views on chronic pain management in Pakistan?
- What are your views regarding current opioid regulation (schedule-G) and access to opioids for the management of CNMP?
- What guidelines and information do you follow while reviewing patient opioid medicine regimens?
- What do you think is needed in the future in the optimization of prescription opioids?

**Opioid medicine related:**

- What is your experience when you review patient’s opioid medicine in CNMP? (prescription related)
- What is your experience when you counsel, educate, and provide medicine information to people suffering from CNMP to optimize their opioid use? (patient oriented)

**Interdisciplinary approaches, health care team related:**

- Do you feel prescribers and community pharmacists can work together as a team to manage chronic pain by opioids?
- How do you feel if a pharmacist has a concern/suggestion about an opioid therapy and usage or suggests a new plan with you?

**“If they highlight positive views about community pharmacy-based pain management and opioid optimisation services), otherwise ask, opinions, views, suggestions or strategies to optimise the use of prescriptions opioids (general)”**

**Perception of the role of community pharmacist in optimization of opioid therapy:**

- Do you think of any services pharmacist could provide for opioid therapy optimization?
- Are community pharmacists playing any role in patient education, counselling, review and monitoring (for misuse and addiction) in prescription opioids in CNMP?
- What do you think the key attributes of care in a service provided by community pharmacist to optimise opioid therapy should be? Any barriers?
- What do you want/expect from a community pharmacist when a CNMP patient goes to fill an opioid prescription?
- What are your current experiences?

“**Ask if there** a**nything they would like to add and highlight which wasn’t addressed during focus group/interview”**

# 4. Interview guide with policy makers

**Health system and pain management:**

- Can you please share your views on current use of opioids in the management of chronic non-malignant pain?
- Can you share your views on chronic pain management in Pakistan?
- What is the reason Pakistan lacks potent opioids, and how can the situation be improves to improve access to medicines? (only ask if they mention lack of opioids, or opioids for cancer pain managements)

**Health care team related:**

- Do you feel prescribers and community pharmacists can work together as a team to manage chronic pain by opioids?

**“If they highlight positive views about community pharmacy-based pain management and opioid optimisation services), otherwise ask, opinions, views, suggestions or strategies to optimise the use of prescriptions opioids (general)”**

**Community pharmacist related:**

- What is your opinion on utilising community pharmacists to avoid opioid prescription misuse and optimise opioid therapy in CNMP to maximise the benefits and reduce harm?
- What is your opinion of current community pharmacist services for CNMP management?

**Opioid medicine related:**

- Where do you see the community pharmacist’s role to help people using prescription opioids in managing CNMP (future role of the community pharmacist)
- What should be the key attributes of a satisfactory review service in community pharmacy to avoid prescription misuse and optimise opioid therapy in CNMP?

“**Ask if there** a**nything they would like to add and highlight which wasn’t addressed during interview”**

**Appendix 2:**

**Checklist for case study observation:**

**Pharmacy related:**

• Was the person received by a pharmacy assistant- pharmacist- called a pharmacist—all dealing with pharmacy staff

• Infrastructure

• Rush hours vs free

• Overall pharmacy opening and closing times- holiday

• Dispensing process

• Number of staff

• Locality or geographical area possible effect

• Timings effect

• Review

• Layout-human factors- to reduce medication errors

• Medicine inventory

• Drug inspector visits

• Medicine entry

• medicine record keeping

• Medicine cabinet

• Ongoing services where staff is engaged

• Anything else

**Personnel related:**

• Pharmacist or pharmacy technician

• Who is receiving orders

• Who is filling prescriptions

• Who reviews

• Who stocks

• Time to dispense

• How many deliveries

• Any telephone contact with the prescriber

• Any telephone contact with the patient

• Anything else

**Questions asked by the pharmacist (all-anything):**

• Any assessment to detect is this prescription for the same patient

• Body language

• Attitude

• Engagement with the patient

• Conversation of the pharmacist

• Duration of time spent with a person filling prescription

• Differences between a new and refill

• Any difference in review or dispensing during consequent refills

• Any difference while giving information to male/female or old/young

• Questions asked if any by the staff or pharmacist

• Questions asked by the patients

• Anything else

**Medicine related- product information- therapeutic goal (people):**

• Feeling after starting a medicine

• Any medicine side effects, problems, concerns

• Do they want any information

• How do they take or use these medicines

• Do they know how to take the medicine

• Do they know signs of overdose and possible course of action

• Lifestyle advice

• Referral

• Information leaflet provided

• Social prescribing

• Any prescription medicine, OTC added

• Any risk establishment- alcohol- sedatives-mental health issue assessment

• Anything else

**Any challenges/barriers/ feelings/concerns expressed by pharmacist or observed: (informal)**

• What is their current experience and Influencers of interaction with CNMP patients taking opioids

• Are they satisfied with the current interaction provided by them in pain management

• Perception about benefit of interaction

• Was the patient approachable

• Any factors/barriers affecting the communication

• Internal: CNMP patient related, personal, knowledge, attitude, time

• External: Policy, guidelines, job description, timings, staffing, remuneration, Doctors, work load

• Medicine review-telephone

• Anything else they added

**Overall reflections:**

**Appendix 3.**

**Table 3: Data saturation and continuation of data collection**

| **Stakeholders** | **Data saturation** | **Number of interviews/focus groups/case studies after data saturation** |
| --- | --- | --- |
| Policy makers | 6^th^ interview | 5 interviews |
| People with CNMP | 9^th^ interview | 5 interviews |
| Doctors | 2^nd^ focus group | 2 focus groups |
| Pharmacists | 3rd focus group | 2 focus groups |
| Case study 1 | 3^rd^ day | 3 days |
| Case study 2 | 2^nd^ day | 4 days |
| Case study 3 | 4^th^ day | 2 days |
| Case study 4 | 3^rd^ day | 3 days |
| Case study 5 | 3^rd^ day | 3 days more |
| Case study 6 | 3^rd^ day | 3 days more |

**Appendix 4**

**Table 4: Demographic information of people with CNMP**

| **Age (years)** | **Education** | **Years of CNMP pain** | **Location** | **Origin of pain** |
| --- | --- | --- | --- | --- |
| 42 | 8^th^ class | 4.5 | Rural | Neuropathic |
| 60 | SSC | 4.5 | Rural | Back pain |
| 52 | HSSC | 1.5 | Urban | Back pain |
| 62 | Graduation | 4.5 | Rural | Road traffic accident |
| 67 | Graduation | 4.5 | Urban | Multiple(not specified) |
| 70 | Graduation | 20 | Urban | Multiple (not specified) |
| 56 | Graduation | 10 | Rural | Knee pain |
| 31 | SSC | 4.5 | Urban | Multiple Sclerosis |
| 70 | Graduation | 2.5 | Urban | Back/neck pain |
| 62 | Graduation | 10 | Urban | Diabetic neuropathy |
| 56 | HSSC | 6 | Rural | Surgical (site not mentioned) |
| 75 | HSSC | 15 | Rural | Knee pain |
| 63 | Graduation | 6 | Urban | Knee pain |
| 59 | Graduation | 1.5 | Urban | Neuropathic |
